# Cationic substitution, dynamical stability, thermal stability, electronic and thermoelectric properties in 2D dialkali metal monoxides via DFT and ML approach

**DOI:** 10.1038/s41598-025-11352-9

**Published:** 2025-07-28

**Authors:** S. Chellaiya, Thomas Rueshwin, R. D. Eithiraj

**Affiliations:** Department of Physics, School of Advanced Sciences, Vellore Institute of Technology (VIT), Chennai, Tamil Nadu 600127 India

**Keywords:** MD simulation, Density functional theory, 2D materials, Alkali-metal oxides, Waste heat recovery, Machine learning, Theory and computation, Two-dimensional materials

## Abstract

Using the WIEN2k software, Density Functional Theory (DFT) was applied to analyse the impact of cationic substitution on the physical features of the 1T-K_2_O monolayer. Phonon dispersion analysis confirmed dynamical stability, Ab-initio Molecular Dynamics (AIMD) simulations validated thermal stability, and cohesive energy calculations ensured thermodynamic stability of 1T-K_2_O. Based on the phonon studies, both 1T-KNaO and 1T-KRbO are dynamically unstable with a slightly visible imaginary frequency. Specifically, for electronic property assessment, generalized gradient approximation (GGA) and hybrid exchange-correlation functionals were utilized. This study unveiled the 1T-KXO (X = Na, K, Rb) monolayers as an indirect band gap semiconductor, for 1T-K_2_O, 1T-KNaO and 1T-KRbO were 0.94 eV (1.84 eV), 1.03 eV (1.94 eV) and 0.84 eV (1.77 eV) obtained implementing GGA and hybrid functionals, respectively. Using a machine learning (ML) approach, the band gap was predicted as 1.45 eV (0.85 eV) for 1T-K_2_O, 1.79 eV (0.97 eV) for 1T-KNaO, and 1.17 eV (0.72 eV) for 1T-KRbO, with random forest regression (linear regression) method. The physical properties were tailored by the impact of cationic substitution on the 1T-K_2_O were studied. The variation in the physical properties were investigated. Optical analysis indicated a strong absorption coefficient, underscoring the 1T-KXO monolayers potential for photovoltaic applications in the UV region. The ZT value obtained at room temperature are 0.58, 0.86 and 0.69 for 1T-K_2_O, 1T-KNaO and 1TKRbO, respectively. Additionally, the 1T-KNaO demonstrated promising thermoelectric properties, at 400 K achieving a figure of merit (ZT) of 0.93, indicating its suitability for waste heat recovery. A ML model was trained to predict the ZT of 1T-KXO using random forest regression and linear regression.

**Supplementary Information:**

The online version contains supplementary material available at 10.1038/s41598-025-11352-9.

## Introduction

During the utilization of fossil fuels, electric energy, and solar energy, a portion of the energy is inevitably dissipated as waste heat. Typically overlooked, this heat energy can have adverse consequences, such as compromising the performance and reliability of logic computing devices and data storage systems. Thermoelectric (TE) devices offer a promising solution by directly converting waste heat into electrical energy, which can either power systems or be utilized for localized cooling, thereby ensuring stable operation. TE materials enable a bidirectional conversion between heat and electricity, making them versatile in energy applications. Furthermore, thermoelectric devices can be made more compact and seamlessly integrated into existing semiconductor chip architectures, functioning as microgenerators or microrefrigerators. Over decades of research, materials such as BiTe-based alloys, SnSe compounds, CuSe compounds, half-Heusler alloys, perovskites, multicomponent oxides, organic-inorganic composites, and GeTe/PbTe systems have demonstrated exceptional optical and thermoelectric properties^1–19^.

Since the groundbreaking discovery of graphene, research on two-dimensional (2D) materials has grown at an unprecedented pace over the past decades. The advent of advanced theoretical simulation techniques and innovative material synthesis methods has led to the prediction and successful fabrication of a diverse range of 2D materials. Prominent examples of two-dimensional materials include graphene, transition metal dichalcogenides (TMDCs), group IVA–VA compounds, nitrides, and MXenes^10,20–27^. The applications of these materials span electronics, optoelectronics, topological spintronics, biomedicine, energy storage, and energy conversion systems, made possible by their remarkable electronic and optical properties across various allotropes. Two-dimensional materials with layered structures have attracted significant attention as potential thermoelectric materials due to their exceptional electronic and mechanical properties. In recent decades, extensive theoretical and experimental studies have focused on materials such as SnSe and MoS_2_^10,22,28–30^. These materials exhibit unique and impressive characteristics, demonstrating great potential for the development of high-performance thermoelectric devices.

Alkali metal oxides have garnered extensive research attention due to their promising applications in energy storage. Among these, Li_2_O has been employed in specialized environments such as spacecraft and submarines for its exceptional ability to absorb CO_2_. Using DFT calculations, it was reported that Li_2_O demonstrates efficient CO_2_ absorption. Similarly, Na_2_O has been utilized to enhance alumina for CO_2_ chemisorption, exhibiting remarkable cyclic stability. In 2007, bulk Li_2_O, Na_2_O, K_2_O, and Rb_2_O were examined theoretically for electronic properties, while their electronic and optical behaviour under pressure was analysed in 2008^31–34^.

Alkali metal oxide two-dimensional (2D) materials have garnered escalating attention in recent years, owing to their significant potential in energy storage and optoelectronic applications. These materials can crystallize in two distinct structural phases known as the 1T phase and the 1 H phase. The crystal structure of 2D transition metal dichalcogenides consists of a hexagonally arranged layer of metal (M) atoms positioned between two layers of chalcogen (X) atoms. In this structure, each chalcogen atom sits at the peak of a triangular pyramid formed by three surrounding metal atoms at the base. The arrangement of chalcogen atoms around each metal atom can exhibit either octahedral or trigonal prismatic symmetry. These configurations are commonly known as the 1T and 1 H phases, respectively. In contrast, for metal oxides, the atomic positions are reversed: the metal atoms occupy the positions typically held by chalcogens, while the oxygen atoms take the place of the metals. A theoretical study on the optoelectronic properties of the 1 H and 1T polymorphs of Li_2_O was performed, revealing that 1 H-Na_2_O exhibits characteristics of a 2D double Weyl semimetal, whereas 1 H-K_2_O behaves as a 2D pseudospin-1 metal. Additionally, 2D alkali metal oxides and their heterostructures were examined for their applicability in excitonic and tandem solar cells. The transport properties of 1 H-Na_2_O and 1 H-Na_2_S were analysed, while the thermoelectric and optical characteristics of 1T-Na_2_O, 1T-Rb_2_O, and 1T-Cs_2_O were systematically investigated. High-efficiency photovoltaic performance is predicted in type-II vdW heterostructures based on the relatively unexplored 1T-phase di-alkali metal oxides and chalcogenides, using DFT-D3 and HSE06 methods. Additionally, tandem solar cell architectures have been explored for these 1T-phase materials^35–41^.

ML has emerged as a powerful tool in TE research, enabling rapid prediction and optimization of key properties of thermoelectric properties. Models like support vector regression, random forests, neural networks, and gradient boosting are commonly used. ML combined with DFT accelerates the identification of high-performance materials, while interpretability methods like SHapley Additive exPlanations offer insights into key descriptors. 2D materials such as ScX, SiX, GeX, SnX, and BX monolayers show excellent TE performance due to quantum confinement, tuneable band gaps, and enhanced phonon scattering. Many (e.g., SnSe, SiSb, BSe) achieve ZT > 1 at high temperatures and exhibit directional anisotropy in transport properties. ML is increasingly used to screen large libraries of 2D materials by learning structure, property relationships, significantly reducing experimental cost and accelerating the discovery of promising thermoelectric materials^42,43^.

This study investigates the impact of cationic substitution on the 1T-K_2_O monolayer. The physical properties of cation-substituted 1T-K_2_O derivatives, namely 1T-KNaO and 1T-KRbO, were systematically analysed. The results reveal that 1T-KXO monolayers exhibit indirect band gaps, suggesting their suitability for photovoltaic applications. A ML model was trained to predict the bandgap and ZT of the 1T-KXO. This work highlights the potential of 1T-KXO monolayers in advancing energy harvesting technologies by providing insights into their physical characteristics.

### Computational details

To investigate the physical properties of the 1T-KXO monolayer, first-principles simulations were performed using WIEN2k, with the full-potential linearized augmented plane wave (FP-LAPW) method derived from density functional theory (DFT)^44,45^. To scrutinize the physical properties of 1T-KXO monolayer, the GGA functional was employed. To accurately predict the bandgap, hybrid functionals (YS-PBE0), were utilized, as GGA was found to underestimate the bandgap^46,47^. Computational parameters such as R_MT_K_MAX_ was set to 7 and a cutoff energy of -6 Ry was applied to distinguish between core and valence states. To mitigate the interlayer interactions, a vacuum of 15 Å was introduced along the z-axis. The Brillouin zone was sampled via a Monkhorst-Pack scheme, with an appropriate k-mesh^48^. For structure optimization, convergence criteria were set as follows: 1 mRy/a.u., 0.0001 eV, and 0.001 e, for forces, energy and charge, respectively, using a 10 × 10 × 1 k-mesh. To verify the dynamical stability of the materials, the phonon spectrum was investigated, and a 2 × 2 × 1 supercell with a 10 × 10 × 1 k-mesh was used. AIMD simulations were undertaken using Siesta software, to assess the thermal stability^49,50^. Using a 2 × 2 × 1 supercell, AIMD simulations were conducted with a time step of 2 fs, running for 3 ps in the NVT ensemble and the annealing temperature was adjusted to 300 K using the Nose thermostat. Physical properties were computed using a 10 × 10 × 1 k-mesh, while for optical properties a 20 × 20 × 1 k-mesh was used. The thermal properties were investigated using the BotlzTraP code integrated with WIEN2k^51^. To train a machine learning (ML) model, linear regression and random forest regression algorithms, implemented via the Scikit-learn library. The training dataset was sourced from the Materials Project database for bandgap prediction and for ZT prediction data available from the literature^52–54^.

### Structural details

**Fig. 1:**
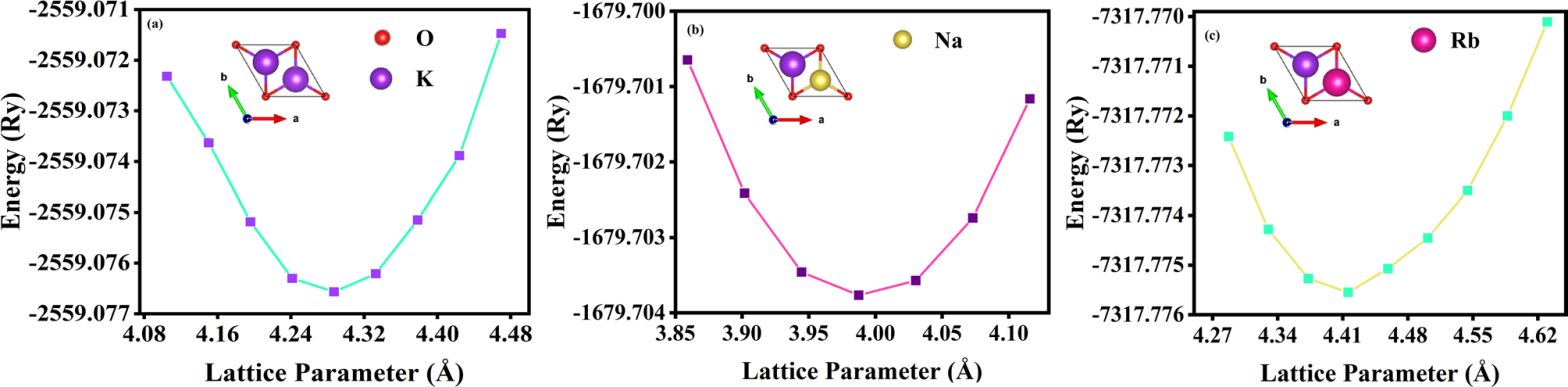
Depicts lattice constant optimization curve of (**a**) 1T-K_2_O, (**b**) 1T-KRbO and (c) 1T-KNaO.

To analyse the structural properties of the 1T-KXO monolayer, the GGA exchange-correlation functional was employed within the DFT method. It was determined that 1T-K_2_O adopts the P-3m1 (164) space group, whereas the 1T-KNaO and 1T-KRbO crystallizes in the space group P3m1 (156). The structural optimization of atomic positions and lattice parameters was a crucial step in achieving the ground-state configuration. It was determined that the optimized lattice parameters (a = b) are 3.98 Å, 4.28 Å and 4.41 Å for 1T-KNaO, 1T-K_2_O and 1T-KRbO, respectively. A vacuum of 15 Å was introduced along the z-axis. The volume optimization curve of 1T-KXO monolayer was illustrated in the Fig. 1.

**Stability**.

To evaluate the thermodynamic stability of the 1T-KXO monolayer, the cohesive energy was calculated. The cohesive energy is determined using the formula^55^:

1 $${\text{E}}_{\text{c}\text{o}\text{h}}=\frac{{\left\{\right(\text{E}}_{\text{K}}+{\text{E}}_{\text{X}}+{\text{E}}_{\text{O}})-{\text{E}}_{\text{K}\text{X}\text{O}}\}}{3}$$

where $\:{\text{E}}_{\text{K}\text{X}\text{O}}$ corresponds to the total energy of 1T-KXO and $\:{\text{E}}_{\text{K}}$, $\:{\text{E}}_{\text{X}}$ and $\:{\text{E}}_{\text{O}}$ are the energies of an isolated K atom, X (X = Na, K, Rb) atom and an O atom, respectively. The computed cohesive energy of 1T-K_2_O, 1T-KNaO, and 1T-KRbO are 3.07 eV/atom, 2.97 eV/atom and 3.06 eV/atom, which aligns closely with other theoretical values^55^. These findings suggest that 1T-KXO possesses a relatively high cohesive energy, indicating its thermodynamic stability. Phonons perform a crucial role in elucidating the dynamic behaviour of materials.

**Fig. 2:**
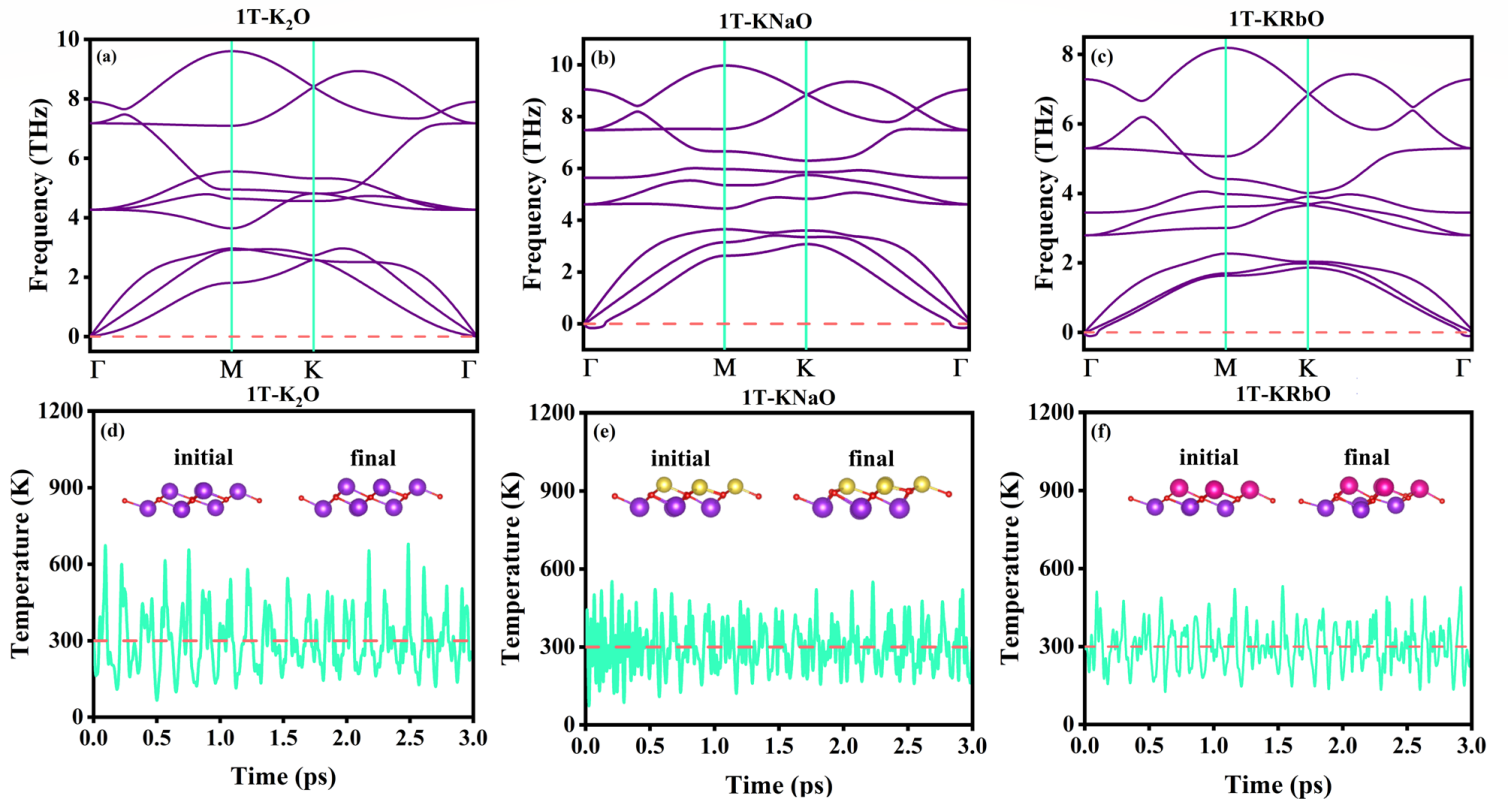
Depicts the phonon dispersion curve of (**a**) 1T-K_2_O, (**b**) 1T-KNaO, (**c**) 1T-KRbO, AIMD calculations of (**d**) 1T-K_2_O, (**e**) 1T-KNaO, and (**f**) 1T-KRbO.

By integrating the Phonopy code with WIEN2k, the phonon dispersion spectrum of 1T-KXO was computed. For these calculations, the 2 × 2 × 1 supercell of 1T-KXO was utilized. As shown in Fig. 2, the phonon dispersion curves reveal no imaginary frequencies (negative values), confirming the dynamic stability theoretically of 1T-K_2_O^39^. The phonon dispersion curves exhibit small imaginary frequencies for 1T-KNaO and 1T-KRbO, indicating dynamic instability. However, both structures can potentially be stabilized through the application of pressure or external strain or by increasing the size of supercell^56^. At room temperature, to assess thermal stability AIMD simulations were conducted using the SIESTA software. A 2 × 2 × 1 supercell of 1T-KXO was annealed at 300 K within the NVT ensemble for a total simulation time of 3 ps, with a time step of 2 fs. The results presented in Fig. 2(d–f) indicate that the 1T-KXO structures remain intact throughout the simulation, confirming the thermal stability of these compounds. The structural integrity of 1T-K_2_O remains nearly unchanged, while 1T-KNaO and 1T-KRbO exhibit slight temperature induced distortions^41^.

### Electronic properties

**Fig. 3:**
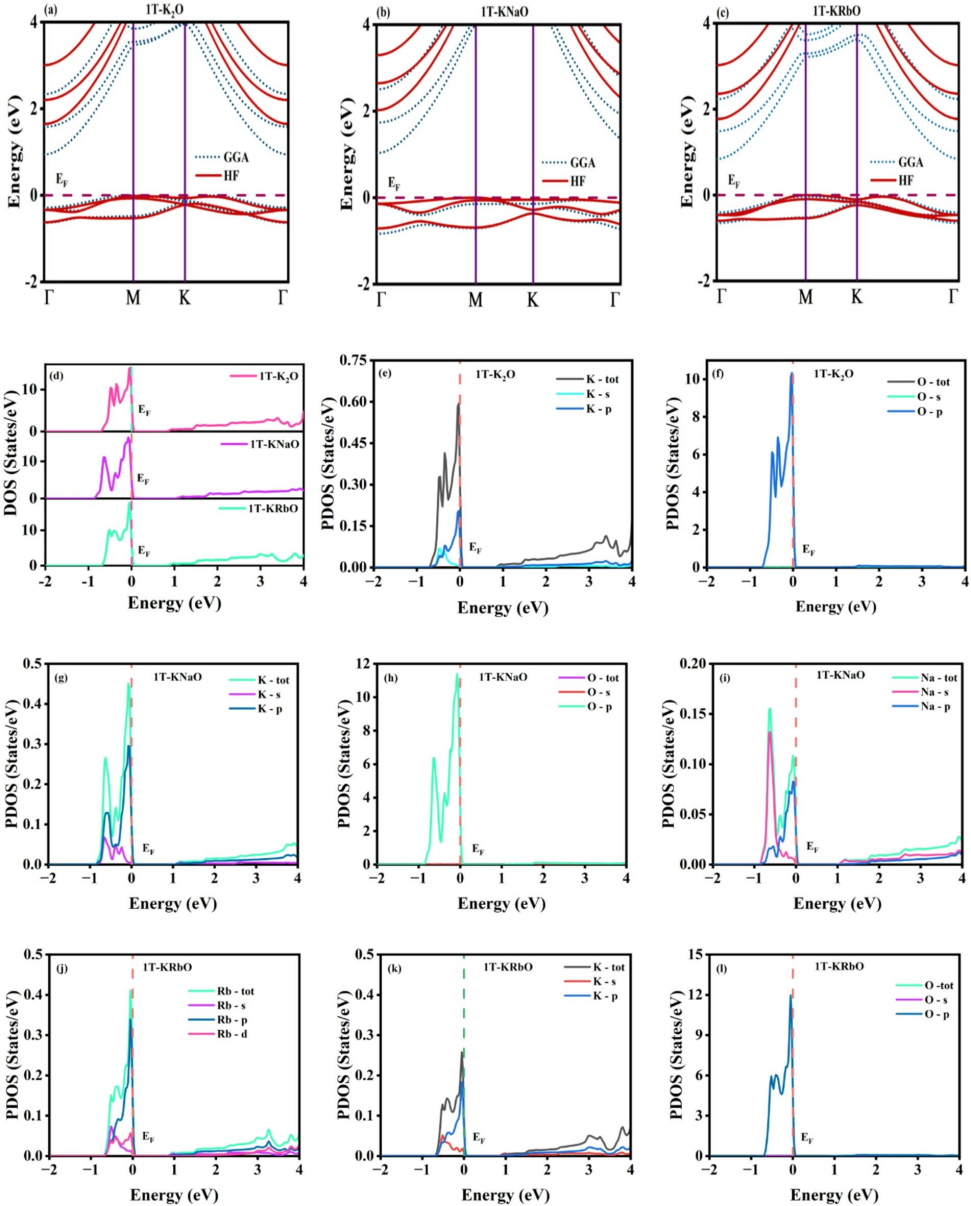
Illustrates band structures of (**a**) 1T-K_2_O, (**b**) 1T-KNaO, (**c**) 1T-KRbO, (**d**) TDOS (total) of 1T-KXO, and (**e**–**l**) PDOS of 1T-KXO.

The electronic properties of 1T-KXO were analyzed in the framework of DFT using WIEN2k^44,45^. The band structure and density of states (DOS) of 1T-KXO were computed to provide detailed insights. Initially, the band structure was determined using the GGA functional, followed by calculations with the hybrid functional YS-PBE0 in WIEN2k, as GGA is known to underestimate band gaps^46,47^. Hybrid functionals are essential for accurate band gap evaluation, especially in the absence of experimental data. The Fermi energy was set to zero to streamline band structure analysis, placing the valence bands in the negative energy range and the conduction band in the positive range. The calculated band gaps for 1T-K_2_O, 1T-KNaO and 1T-KRbO were 0.94 eV (1.84 eV), 1.03 eV (1.94 eV) and 0.84 eV (1.77 eV) using GGA (hybrid) functionals, respectively. A clear size effect is observed, wherein an increase in cation size leads to a reduction in the bandgap. The calculated band gaps of 1T-K_2_O are 0.94 eV (GGA) and 1.84 eV (hybrid), which differ from the previously reported value of 1.25 eV, likely due to variations in computational parameters such as pseudopotentials and atomic positions. As illustrated in Fig. 3(a-c), 1T-KXO is identified as an indirect bandgap semiconductor across both methods, with the valence band maximum (VBM) located at the high-symmetry k-point M and the conduction band minimum (CBM) at Γ. The DOS, encompassing both TDOS (total) and PDOS (projected), was further analysed, to develop a thorough insight of the electronic properties, as depicted in Fig. 3(d-l). The Fermi energy (E_F_) was aligned at 0 eV, with the valence band situated below and the conduction band above this reference. The PDOS analysis of the 1T-KXO compounds provides insight into the elemental contributions to the electronic states. In all cases, oxygen (O) p-orbitals predominantly define the valence band. For 1T-K_2_O, potassium (K) significantly contributes to the conduction band. In 1T-KNaO, both K and sodium (Na) participate in the conduction band, with K having a greater influence. Similarly, in 1T-KRbO, both K and rubidium (Rb) contribute to the conduction band, with K playing a dominant role. Furthermore, the band gap values obtained from the DOS calculations align well with those derived from the band structure, reinforcing the reliability of the results.

### Optical properties

The potential of the 1T-KXO monolayer for optoelectronic applications was scrutinized using computational approaches within the WIEN2k framework. The fundamental optical properties were explored using the GGA functional. The optical properties were mathematically described^57–59^ as follows

2 $$\text{A}\text{b}\text{s}\text{o}\text{r}\text{p}\text{t}\text{i}\text{o}\text{n}\:\text{c}\text{o}\text{e}\text{f}\text{f}\text{i}\text{c}\text{i}\text{e}\text{n}\text{t},\:\alpha\:\left(\omega\:\right)=\sqrt{2}\omega\:{({\left[{\epsilon\:}_{1}^{2}-{\epsilon\:}_{2}^{2}\right]}^{2}-{\epsilon\:}_{1})}^{1/2}$$

3 $$\text{R}\text{e}\text{f}\text{r}\text{a}\text{c}\text{t}\text{i}\text{v}\text{e}\:\text{i}\text{n}\text{d}\text{e}\text{x},\:n\left(\omega\:\right)=\:\frac{1}{\sqrt{2}}{\left[{\left\{{\epsilon\:}_{1}^{2}\left(\omega\:\right)+{\epsilon\:}_{2}^{2}\left(\omega\:\right)\right\}}^{1/2}+{\epsilon\:}_{1}\left(\omega\:\right)\right]}^{1/2}$$

4 $$\text{R}\text{e}\text{f}\text{l}\text{e}\text{c}\text{t}\text{i}\text{v}\text{i}\text{t}\text{y},\:R\left(\omega\:\right)=\:{\left|\frac{\sqrt{{\epsilon\:}_{1}\left(\omega\:\right)+{i\epsilon\:}_{2}\left(\omega\:\right)}-1}{\sqrt{{\epsilon\:}_{1}\left(\omega\:\right)+{i\epsilon\:}_{2}\left(\omega\:\right)}+1}\right|}^{2}$$

5 $$\text{E}\text{n}\text{e}\text{r}\text{g}\text{y}\:\text{L}\text{o}\text{s}\text{s},\:L\left(\omega\:\right)=\:-Im\:\left(\frac{1}{\epsilon\:\left(\omega\:\right)}\right)$$

**Fig. 4:**
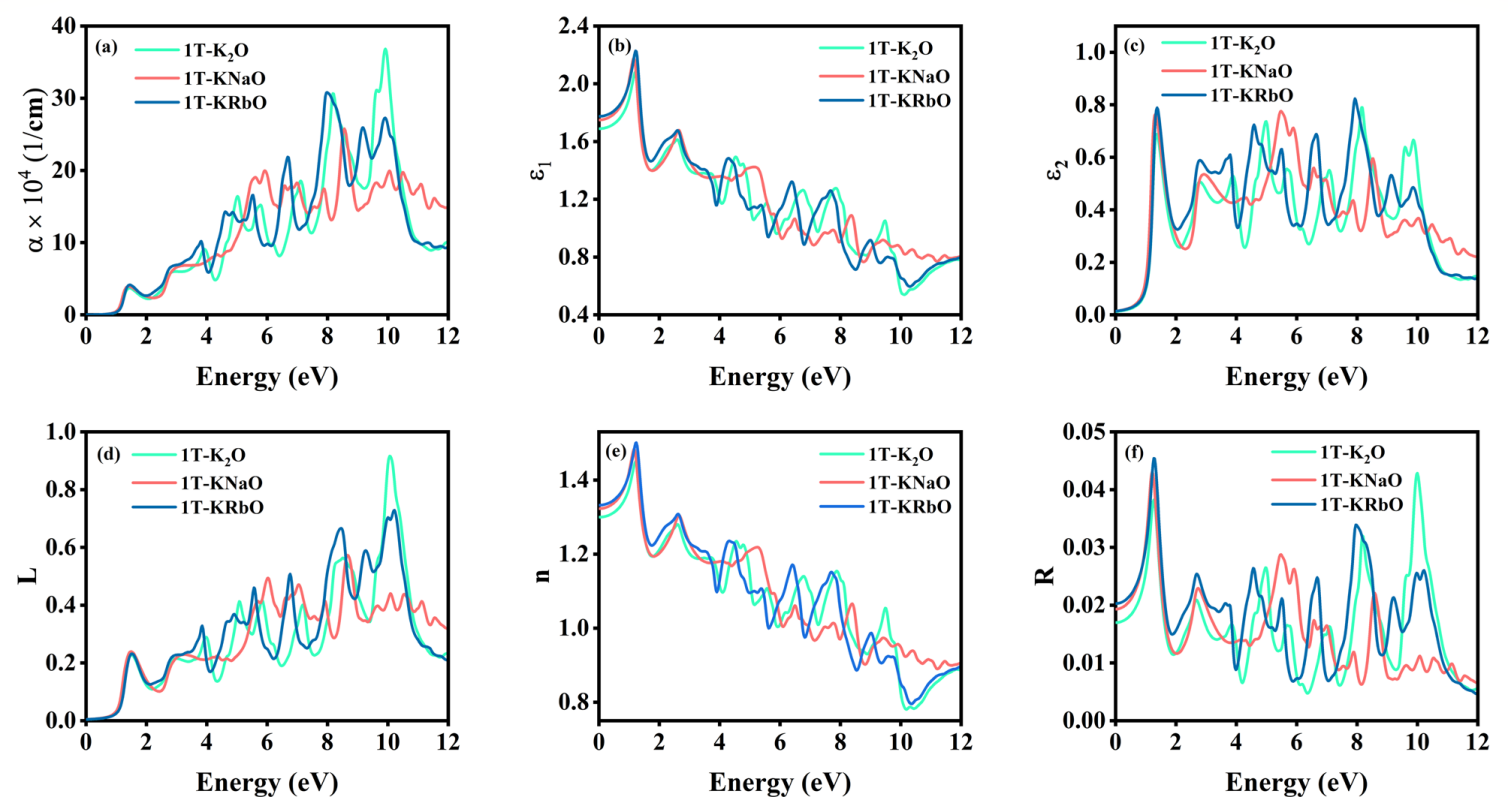
Illustrates the (**a**) absorption coefficient, (**b**) dielectric function real part, (**c**) dielectric function imaginary part, (**d**) Energy Loss, (**e**) Refractive index, (**f**) Reflectivity of 1T-KXO.

The complex dielectric function encapsulates a material’s interaction with electromagnetic fields, expressed as $\:{\upepsilon\:}\left({\upomega\:}\right)={{\upepsilon\:}}_{1}\left({\upomega\:}\right)+\text{i}{{\upepsilon\:}}_{2}\left({\upomega\:}\right)$, where the $\:{{\upepsilon\:}}_{1}\left({\upomega\:}\right)$ is associated with the material’s polarizability and the $\:{{\upepsilon\:}}_{2}\left({\upomega\:}\right)$ is associated with optical absorption^60^. Figure 4 (a-f) the optical response of 1T-KXO. The static dielectric constants were found as 1.69, 1.75 and 1.77 for 1T-K_2_O, 1T-KNaO and 1T-KRbO, respectively. The optimum peak values of $\:{{\upepsilon\:}}_{1}\left({\upomega\:}\right)$ were found in the visible region for 1T-K_2_O, 1T-KNaO and 1T-KRbO were 2.08 (1.21 eV), 2.18 (1.16 eV) and 2.23 (1.21 eV). After this, the $\:{{\upepsilon\:}}_{1}\left({\upomega\:}\right)$ decreases and keep on oscillating throughout the energy range. $\:{{\upepsilon\:}}_{2}\left({\upomega\:}\right)$ is linked to inter and intra-band transitions, as the excitation from the occupied level to the unoccupied level. The optimum peak appears in the visible region are 0.689 (1.37 eV), 0.77 (1.32 eV) and 0.79 (1.37 eV), respectively. The absorption coefficient indicates how much light is absorbed by a material over a given distance. Before the bandgap, 1T-KXO exhibits negligible absorption. Beyond the bandgap, absorption increases; though absorption peaks occur in the UV range, practical applications favour visible-light absorption for photovoltaic devices. While strong UV absorption suggests the potential of these materials for applications in UV photodetectors, UV shielding, or sterilization devices, their comparatively weaker absorption in the visible range limits their immediate applicability in visible-light-driven optoelectronic and photovoltaic devices, which require efficient solar spectrum harvesting. The first peak appears for 1T-K_2_O is $\:3.70\:\times\:\:{10}^{4}\:{\text{c}\text{m}}^{-1}$ (1.43 eV), for 1T-KNaO is $\:3.94\:\times\:\:{10}^{4}\:{\text{c}\text{m}}^{-1}$ (1.40 eV) and 1T-KRbO is $\:4.14\:\times\:\:{10}^{4}\:{\text{c}\text{m}}^{-1}$ (1.46 eV). $\:L\left(\omega\:\right)$, representing the material’s response to photon energy, remains negligible up to the bandgap but peaks at ultra violet region, indicating energy dissipation during absorption. In the visible region, the $\:L\left(\omega\:\right)$ is less indicating most of the light absorbed is not lost. The $\:n\left(\omega\:\right)$ measures the bending of light, with a static value for 1T-K_2_O, 1T-KNaO and 1T-KRbO are 1.29, 1.32, and 1.33, respectively. The maximum $\:n\left(\omega\:\right)$, 1.45 (1.21 eV), 1.48 (1.16 eV) and 1.50 (1.24 eV), observed for 1T-K_2_O, 1T-KNaO and 1T-KRbO, respectively. $\:R\left(\omega\:\right)$ provides insights into the material’s surface behaviour toward light. 1T-K_2_O has maximum peak in the ultra violet region, the cationic effect reduces it, which can be observed in the Fig. 4 (f), as the optimum value for 1T-KRbO and 1T-KNaO are in the visible region. The first peak values for 1T-K_2_O, 1T-KNaO and 1T-KRbO are 0.038 (1.26 eV), 0.043 (1.21 eV), and 0.045 (1.26 eV), respectively. Based on the results of the optical properties, it can be observed that cationic substitution effectively tunes these properties. These findings emphasize the possibilities of the 1T-KXO monolayer as a promising material for optoelectronic applications, particularly in the visible and UV ranges.

### Thermoelectric properties

The ongoing energy scarcity and growing environmental threats necessitate a shift from fossil fuels to sustainable energy solutions, fostering the development of technologies such as solar cells and wind turbines. Nevertheless, a considerable amount of generated energy is lost in the form of waste heat. Despite the overall efficiency of recovery systems remains relatively low, thermoelectric materials and devices offer a promising route to harness this wasted energy^10^. Investigating the thermoelectric properties of materials is crucial for improving device modelling and performance. Thermoelectric parameters were computed using a combination of BoltzTraP and WIEN2k, along with their mathematical formulations^61,62^ as follows:

6 $${{\upsigma\:}}_{{\upalpha\:}{\upbeta\:}}\left(\text{T};{\upmu\:}\right)=\:\frac{1}{{\Omega\:}}\int\:{{\upsigma\:}}_{{\upalpha\:}{\upbeta\:}}\left({\upepsilon\:}\right)\left[-\frac{{\partial\:\text{f}}_{{\upmu\:}}\left(\text{T};{\upepsilon\:}\right)}{\partial\:{\upepsilon\:}}\right]\text{d}{\upepsilon\:}$$

7 $${{\upkappa\:}}_{{\upalpha\:}{\upbeta\:}}^{0}\left(\text{T};{\upmu\:}\right)=\:\frac{1}{{\text{e}}^{2}\text{T}{\Omega\:}}\:\int\:{{\upsigma\:}}_{{\upalpha\:}{\upbeta\:}}\left({\upepsilon\:}\right){\left({\upepsilon\:}-\:{\upmu\:}\right)}^{2}\left[-\frac{{\partial\:\text{f}}_{{\upmu\:}}\left(\text{T};{\upepsilon\:}\right)}{\partial\:{\upepsilon\:}}\right]\text{d}{\upepsilon\:}$$

8 $$\text{S}\text{e}\text{e}\text{b}\text{e}\text{c}\text{k}\:\text{c}\text{o}\text{e}\text{f}\text{f}\text{i}\text{c}\text{i}\text{e}\text{n}\text{t},\:\text{S}=\:\frac{{\Delta\:}\text{V}}{{\Delta\:}\text{T}}$$

9 $$\:\:\:\:\:\:\:\:\:\:\:\:\:\:\:\:\:\:\:\:\:\:\:\:\:\:\:\:\:\:\:\:\:\:\:\:\:\:\:\:\:\:\:\:\:\:\:\:\:\:\:\:\text{P}\text{F}=\:{\text{S}}^{2}{\upsigma\:}\:\:\:\:\:\:\:\:\:\:\:\:\:\:\:\:\:\:\:\:\:\:\:\:\:\:\:\:\:\:\:\:\:\:\:\:\:\:\:\:\:\:\:\:\:\:\:\:\:\:\:\:\:\:\:\:\:\:\:\:\:\:\:\:\:\:\:\:\:\:\:\:\:\:\:\:\:\:\:\:\:\:\:\:\:\:\:\:\:\:\:\:\:\:\left(9\right)$$

10 $$\text{Z}\text{T}=\:\frac{{\text{S}}^{2}{\upsigma\:}\text{T}}{{\kappa\:}_{e}+{\kappa\:}_{l}}$$

**Fig. 5:**
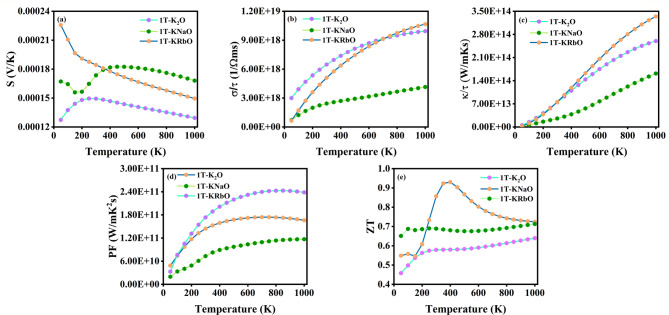
Illustrates the thermoelectric properties such as (**a**) electrical conductivity, (**b**) thermal conductivity, (**c**) Seebeck coefficient, (**d**) Power factor and (e) Figure of merit of 1T-KXO.

Fermi–Dirac distribution function is denoted by f_µ_. The electrical conductivity ($\:{\upsigma\:}$) and electronic thermal conductivity ($\:{\upkappa\:}$_e_) are represented with independent components α and β, while T, $\:\epsilon\:$ and $\:{\upmu\:}$ denote temperature, energy, and chemical potential, respectively. The values of these properties for 300 K and the optimum values are tabulated in Table 1. High and low are desirable quarks for efficient thermoelectric materials as they reduce Joule heating. The motion of charge carriers from regions of high to low temperature, which facilitates current flow, is governed by electrical conductivity. Thermal conductivity, on the other hand, involves heat transfer through both free charge carriers (electronic part) and lattice vibrations (lattice part). The cationic substitution in 1T-K_2_O reduces the electrical conductivity at low temperatures for both 1T-KRbO and 1T-KNaO. Figure 5 (a-e) illustrates the thermoelectric properties of 1T-KXO. It can be observed that 1T-KRbO possesses high electrical conductivity at higher temperatures. The size effect of the atom is evident in the electronic thermal conductivity plot in Fig. 5(c), where 1T-KRbO exhibits a higher value than the others across most temperature ranges. The S, which quantifies the voltage developed across a temperature gradient, was positive throughout the temperature range, confirming that 1T-KXO monolayers are p-type semiconductors. 1T-K_2_O exhibits the lowest S throughout the temperature range. At low temperatures, 1T-KRbO has a higher S than 1T-KNaO. However, at high temperatures, 1T-KNaO exhibits the highest S. The thermoelectric performance evaluated by the power factor follows the order shown in Fig. 5(d), with 1T-KRbO being the highest, followed by 1T-K_2_O, and then 1T-KNaO. The size effect plays a crucial role here because as the size of the atom increases, the thermoelectric performance also improves. ZT was calculated using both the lattice and electronic part of the thermal conductivity. The lattice thermal conductivity ($\:{\kappa\:}_{l}$) can be found using slack Eqs.^63,64^ are given by,

11 $${\kappa\:}_{l}=A\frac{{\stackrel{-}{M}}_{a}{\delta\:\theta\:}_{a}^{3}}{{\gamma\:}^{2}{Tn}^{2/3}}$$

$\:\gamma\:$ is the Gr$\:\ddot{u}$neisen parameter, and can be determined by, $\:\gamma\:=\:\frac{9-12{\left(\frac{{v}_{t}}{{v}_{l}}\right)}^{2}}{2+4{\left(\frac{{v}_{t}}{{v}_{l}}\right)}^{2}}\:$. $\:A$ is the physical parameter and represented as $\:A=\:\frac{2.43\times\:{10}^{-8}}{1-\:\frac{0.514}{\gamma\:}+\frac{0.288}{{\gamma\:}^{2}}}$. Mass per atom is represented as $\:{\stackrel{-}{M}}_{a}$, representing the cube root ratio of a single atom’s volume, $\:\delta\:$, alongside n, which denotes the number of atoms in a unit cell, and T, which specifies the temperature, are key parameters, transverse and longitudinal velocities are given by $\:{v}_{t}$ and $\:{v}_{l}$, $\:{\theta\:}_{a}$ is the acoustic Debye temperature. The relation between the $\:{\theta\:}_{a}\:$and $\:{\theta\:}_{e}$ (the elastic Debye temperature) is given by $\:{\theta\:}_{a}$ = $\:{\theta\:}_{e}{n}^{-1/3}$. Bi_2_Te_3_, a commonly utilized thermoelectric material, exhibits a ZT value of 0.8^65^. Among the 1T-KXO compounds, 1T-KNaO exhibits the highest ZT value of 0.93 at 400 K, whereas 1T-K_2_O demonstrates the lowest. The ZT value of 1T-KNaO achieved by are competitive, underscoring its promise as a viable alternative to traditional thermoelectric materials for energy harvesting applications. The thermal property results suggest that cationic substitution has a significant influence on tuning these characteristics.

**Table 1:** Summarizes the thermoelectric properties at 300 K along with their corresponding optimum values.

Properties	$\:\mathbf{S}\times\:{10}^{-4}$ $\:(\mathbf{V}/\mathbf{K})$	$\:\varvec{\sigma\:}\:\times\:{10}^{18}$ $\:(1/\varvec{\Omega\:}\mathbf{m}\mathbf{s})$	$\:\varvec{\kappa\:}\:\times\:{10\:}^{13}$ $\:(\varvec{W}/\varvec{m}\varvec{K}\varvec{s})$	$\:\varvec{P}\varvec{F}\:\times\:{10}^{10}$ (W/mK^2^s)	ZT
1T-K_2_**O**	1.49 (300 K) 1.50 (250 K)	6.47 (300) 9.94 (1000)	7.49 (300) 25.99 (1000)	14.44 (300) 17.42(700)	0.58 (300) 0.64 (1000)
1T-KNaO	1.74 (300 K) 1.82 (450 K)	2.43 (300) 4.15 (1000)	2.56 (300) 16.17 (1000)	7.32 (300) 11.70(1000)	0.86 (300) 0.93 (400)
1T-KRbO	1.84 (300 K) 2.26 (50 K)	5.11 (300) 10.67 (1000)	7.56 (300) 33.43 (1000)	17.35 (300) 24.30 (850)	0.69 (300) 0.71 (1000)

### Carrier mobility

Efficient thermo-energy generation critically depends on the effective charge separation and retention of high carrier mobility. Elevated mobility and rapid charge transfer are intrinsically linked to a reduced effective mass of charge carriers. The effective mass ($\:{\text{m}}^{\text{*}}$) is mathematically expressed^65^ as

12 $${\text{m}}^{{\text{*}}} = \frac{{\hbar \:^{2} }}{{\frac{{\partial \:^{2} {\text{E}}}}{{\partial \:{\text{k}}^{2} }}}}$$.

where $\:\text{k}$ represents the wave vector, E is the energy, and $\hbar$ is the reduced Planck constant. The deformation potential theory, applied within the effective mass approximation, was used to determine carrier mobility, $\:\mu\:=\:\frac{{e\hslash\:}^{3}{C}_{2D}}{{k}_{B}T{\left({m}^{*}\right)}^{2}{E}_{1}^{2}}$. where $\:{C}_{2D}$ is the elastic modulus in 2D, and $\:{E}_{1}$ is the deformation potential. The $\:{C}_{2D}$ is represented as $\:{C}_{2D}=\frac{1}{{S}_{0}}\frac{{\partial\:}^{2}E}{{\partial\:\epsilon\:}^{2}}$. Here, $\:\epsilon\:$ is the applied strain expressed as $\:\epsilon\:=\:\frac{\varDelta\:a}{{a}_{0}}$. The $\:{E}_{1}$ is calculated using, $\:{E}_{1}=\:\frac{{\partial\:E}_{edge}}{\partial\:\epsilon\:}$. Additionally, the momentum relaxation time ($\:\tau\:$) was derived using the relation, $\:\tau\:=\:\frac{{m}^{*}\mu\:}{e}$^65^. The computed values for these parameters are presented in Table 2. These results provide valuable insights into the interplay between effective mass, mobility, and recombination, essential for optimizing thermoelectric materials.

**Table 2:** Represents the $\:{\text{m}}^{\text{*}}$, $\:{\text{C}}_{2\text{D}}$, $\:{E}_{1}$, and $\:\mu\:$ of charge carriers.

Material	Properties	$\:{\mathbf{m}}^{\mathbf{*}}\:({\mathbf{m}}_{\mathbf{e}}$)	C_2D_(eV/Å)^2^	$\:{\mathbf{E}}_{1}\left(\mathbf{e}\mathbf{V}\right)$	$\:\varvec{\upmu\:}\left({\mathbf{c}\mathbf{m}}^{2}{\mathbf{V}}^{-1}{\mathbf{s}}^{-1}\right)$	$\:\varvec{\tau\:}$ (10^− 14^ s)
1T-K_2_**O**	Holes	1.21	5.00	5.42	39.19	2.70
	Electrons	0.30	5.00	6.73	406.60	7.00
1T-KNaO	Holes	1.21	5.75	4.75	58.63	4.04
	Electrons	0.30	5.75	5.87	614.39	10.58
1T-KRbO	Holes	1.51	5.02	4.28	40.32	3.47
	Electrons	0.30	5.02	4.63	862.71	14.86

### Machine learning

Machine learning (ML) provides an efficient alternative to conventional DFT methods for predicting band gaps, allowing for rapid screening of materials at a much lower computational cost. Nevertheless, the effectiveness of ML models is strongly influenced by the quality of input features and training data. In this work, an ML model was developed and validated using basic geometric and compositional features to predict the band gaps of inorganic materials. In this study, a ML model was developed using linear regression and random forest regression algorithms, both implemented via the Scikit-learn software package^52^. The training dataset for band gap values was sourced from the Materials Project database, while ZT data were obtained from the literature^53,54^. Random forest regression, an ensemble learning technique based on bagged decision trees, is known for its computational efficiency, reliable accuracy, and robust performance in handling large, high-dimensional datasets^52^. In this study, the band gaps of 1T-KXO were predicted using two machine learning models, Random Forest and Linear Regression. The investigation specifically focused on the compounds 1T-K₂O, 1T-KRbO, and 1T-KNaO. Simple geometric and compositional features such as atomic number statistics, lattice parameters, and interatomic distances were extracted from their crystal structures and used as input for the models.

The dataset for training was curated from the Materials Project database, including inorganic materials with band gaps ranging from 0.1 eV to 2 eV. Two regression models, Random Forest and Linear Regression, were trained on this dataset using an 80/20 train-test split to assess performance. The analysis was conducted in two phases: first on the full dataset, and then on a filtered subset containing only oxygen-containing materials. Model performance was evaluated using standard metrics, including Root Mean Square Error (RMSE) and the coefficient of determination (R^2^).

For the targeted 1T-KXO compounds, the Random Forest model predicted band gaps of 1.07 eV for 1T-K_2_O, 0.91 eV for 1T-KNaO, and 0.91 eV for 1T-KRbO, values that are reasonably close to those obtained from density functional theory (DFT) calculations. The Linear Regression model yielded slightly higher predictions for 1T-K_2_O, 1T-KNaO, and 1T-KRbO of 1.01 eV, 1.00 eV, and 1.05 eV, respectively. These were compared with DFT-calculated band gaps using GGA functionals are 0.94 eV for 1T-K_2_O, 1.03 eV for 1T-KNaO, and 0.84 eV for 1T-KRbO. Both machine learning models produced band gap predictions in close proximity to the DFT values, indicating reasonable accuracy. However, the expected size effect typically observable through systematic trends in band gaps was not clearly reflected in the predictions. This discrepancy could be attributed to limitations in the feature set, which may not fully capture the structural and electronic complexities influencing the band gap. While both models showed comparable results, the Random Forest model exhibited better alignment with DFT values, owing to its ability to model nonlinear relationships and complex feature interactions. In contrast, the Linear Regression model, constrained by its assumption of linearity, demonstrated a tendency to slightly overpredict the band gaps.

In addition to band gap prediction, a separate machine learning framework was developed to estimate the thermoelectric figure of merit (ZT) for 1T-KXO compounds across a range of temperatures. The dataset for this task, comprising 4750 entries, was obtained from the supplementary materials of a published research article and included thermoelectric properties such as the Seebeck coefficient, electrical conductivity, and thermal conductivity^54^. Both Random Forest and Linear Regression models were trained using an 80/20 train-test split. To further test generalizability, the models were also evaluated on a subset containing only alkali metal oxides, specifically, 1T-K_2_O, 1T-KNaO, and 1T-KRbO.

**Fig. 6:**
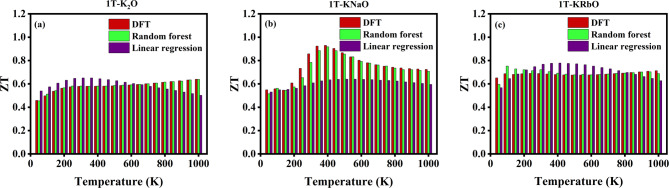
Illustrates the ZT values obtained from DFT calculations, the Random Forest model, and the Linear Regression model.

The predicted ZT values were compared with those obtained from DFT calculations over various temperatures. As illustrated in Fig. 6, the Random Forest model consistently delivered predictions that closely matched the DFT results, affirming its robustness and suitability for modelling temperature-dependent thermoelectric performance in complex oxide systems. The detailed values were tabulated in the supplementary section table s4.

## Conclusion

In this study, the impact of cationic substitution on the physical characteristics of the 1T-K_2_O was systematically explored using DFT within the WIEN2k framework. The dynamical, thermal, and thermodynamic stability of the 1T-KXO (X = Na, K, Rb) monolayers was confirmed through phonon dispersion analysis, Ab-initio Molecular Dynamics (AIMD) simulations, and cohesive energy calculations, respectively. The electronic structure analysis revealed an indirect band gap nature, with band gaps of 0.94 eV (1.84 eV), 1.03 eV (1.94 eV), and 0.84 eV (1.77 eV) for 1T-K_2_O, 1T-KNaO, and 1T-KRbO, respectively, using GGA and hybrid functionals. A ML model was developed to predict the band gap, yielding values of 1.07 eV (1.01 eV), 0.91 eV (1.00 eV) and 0.91 eV (1.05 eV) random forest regression (linear regression) for 1T-K_2_O, 1T-KNaO, and 1T-KRbO, respectively. Furthermore, cationic substitution significantly tailored the physical properties of the monolayers. Optical analysis highlighted a high absorption coefficient, suggesting the 1T-KXO monolayers as promising candidates for optoelectronic applications in the UV region. The thermoelectric properties demonstrated enhanced performance, with ZT values of 0.58, 0.69, and 0.86 at 300 K for 1T-K_2_O, 1T-KNaO, and 1T-KRbO, respectively. Notably, 1T-KRbO exhibited superior thermoelectric efficiency, attaining a ZT value of 0.93 at 1000 K, indicating its potential for waste heat recovery applications. A machine learning model was further employed to predict ZT values using random forest regression and linear regression, reinforcing the reliability of computational approaches in materials design. These findings underscore the tuneable electronic, optical, and thermoelectric properties of 1T-KXO monolayers, establishing them as promising candidates for next-generation optoelectronic and thermoelectric applications.

## Electronic supplementary material

Below is the link to the electronic supplementary material.

Supplementary Material 1

## Data Availability

The datasets used and/or analysed during the current study available from the corresponding author on reasonable request.
