# Supplementary material for: Cationic substitution, dynamical stability, thermal stability, electronic and thermoelectric properties in 2D dialkali metal monoxides via DFT and ML approach
Source: Sci Rep. 2025 Jul 28;15:27514. doi: 10.1038/s41598-025-11352-9 (PMC12304166; doi:10.1038/s41598-025-11352-9)
Supplement: Supplementary file 1 — Supplementary Material 1 [file 41598_2025_11352_MOESM1_ESM.docx]

**Supplementary Section**

**Thermal stability:**


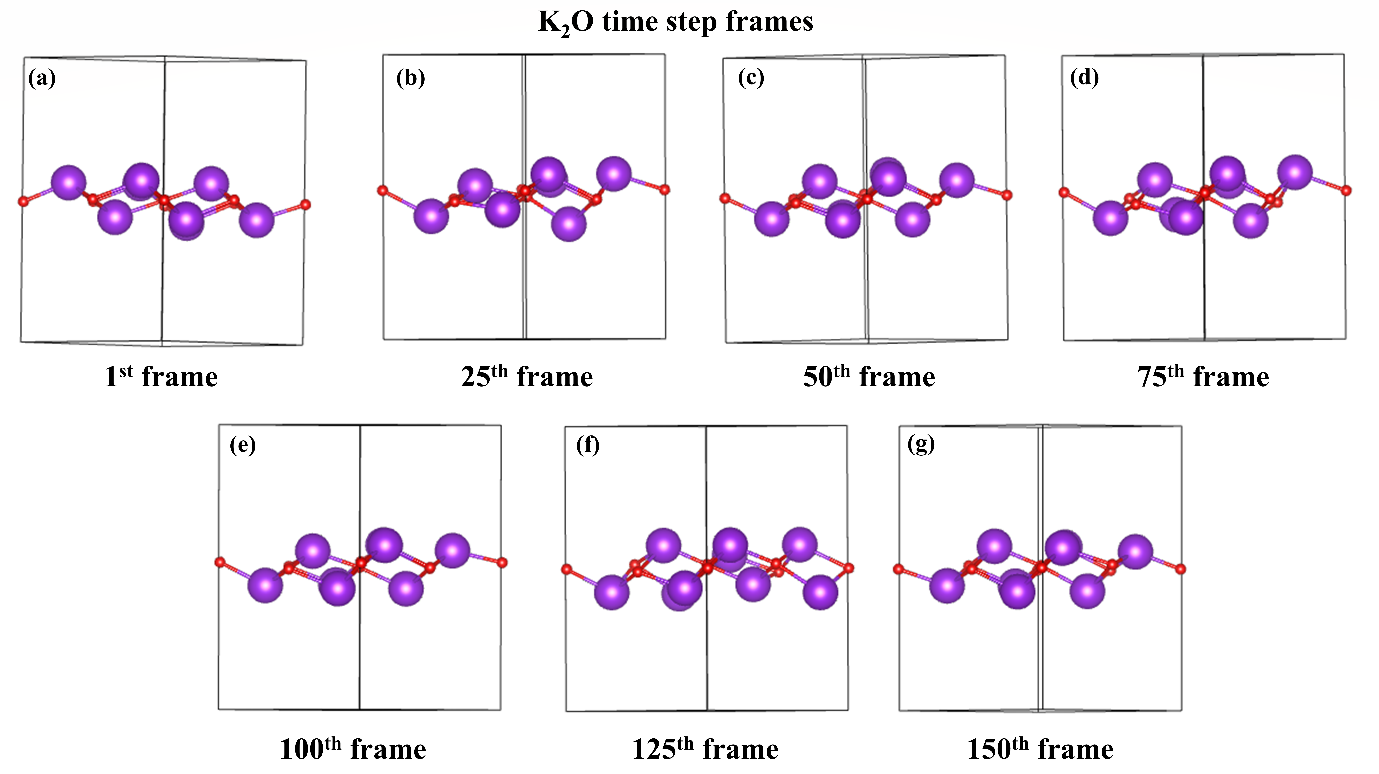


Fig S1. Illustrates the time step frames of AIMD calculation of 1T-K_2_O, where violet ball represents K and red ball represents O.


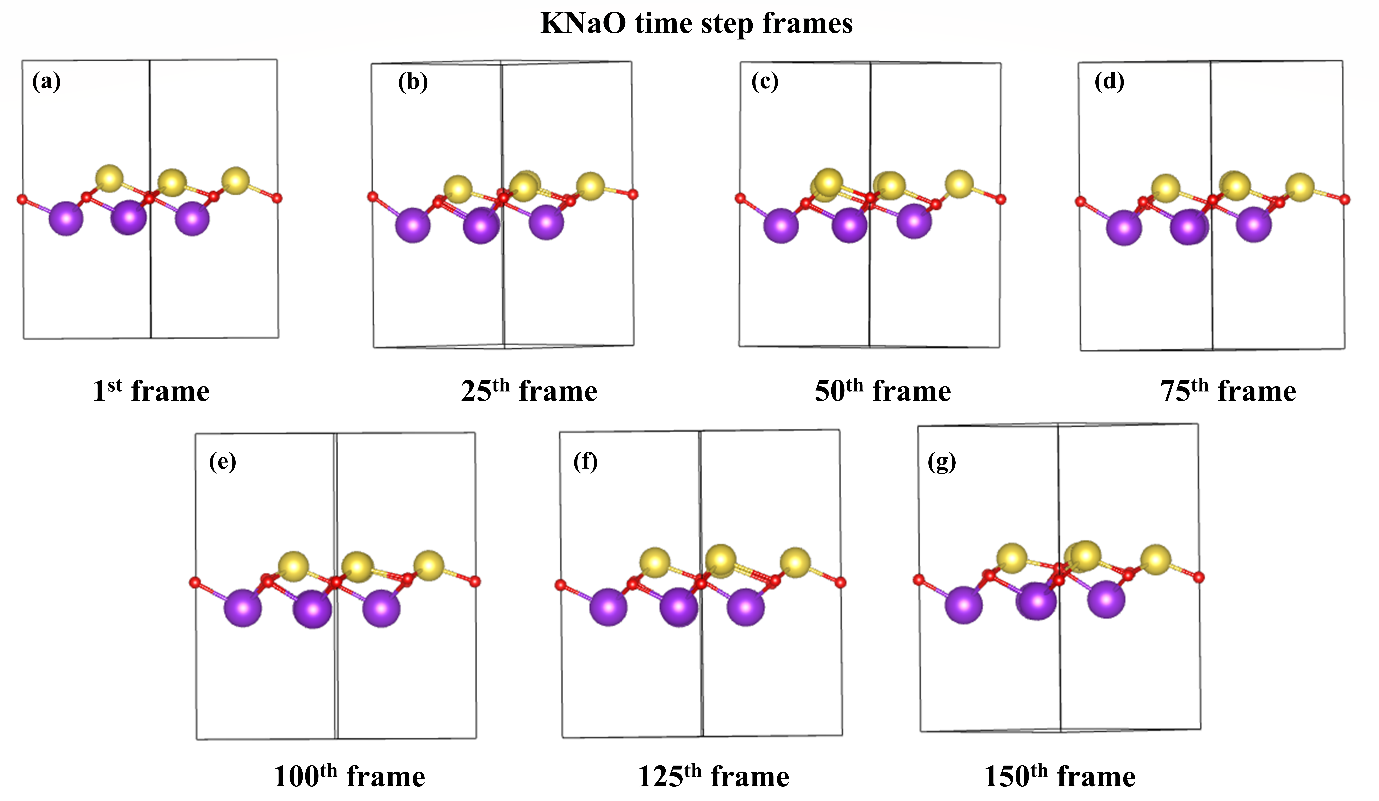


Fig S2. Illustrates the time step frames of AIMD calculation of 1T-KNaO, where violet ball represents K, yellow ball represents Na and red ball represents O.


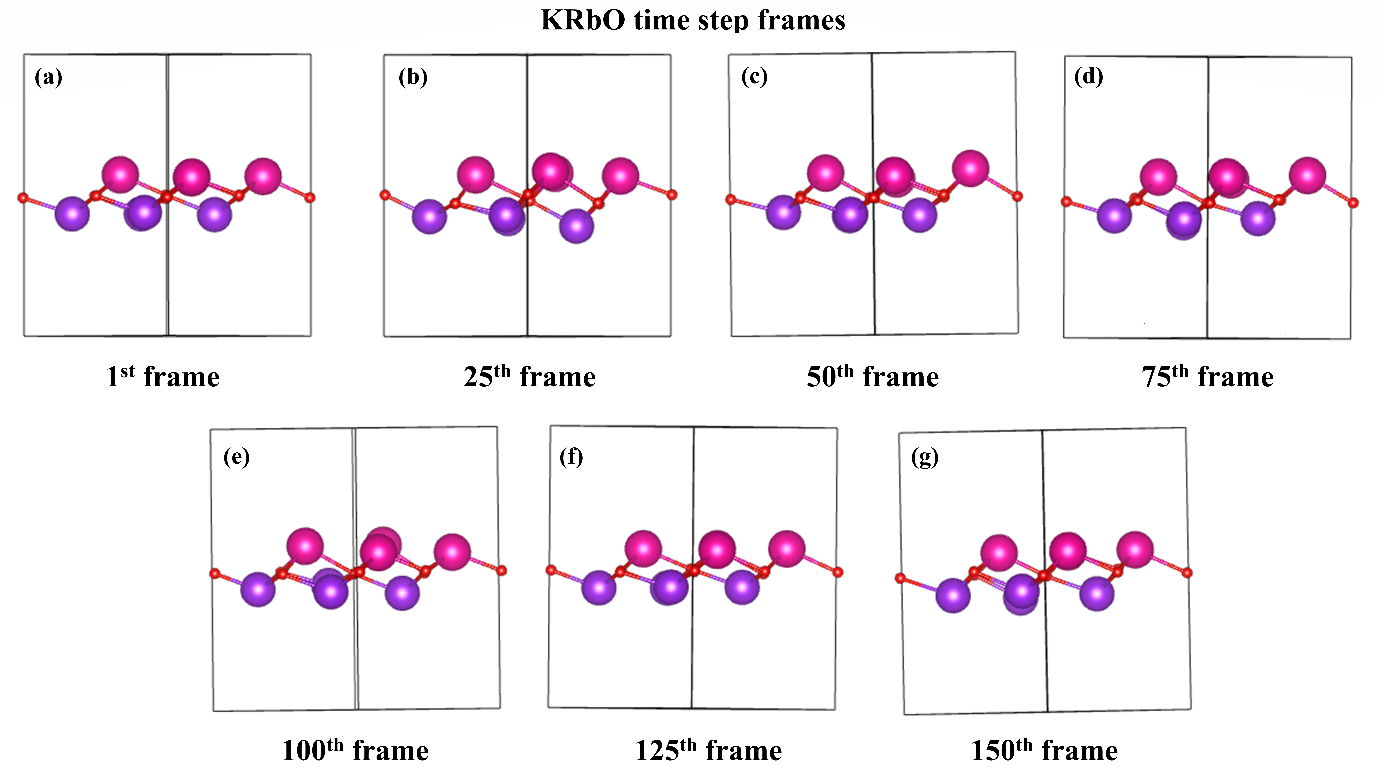


Fig S3. Illustrates the time step frames of AIMD calculation of 1T-KRbO, where violet ball represents K, pink ball represents the Rb and red ball represents O.

**Electronic Property:**

The main electronic structure calculations were conducted using WIEN2k without explicit inclusion of van der Waals corrections, due to technical limitations associated with implementing DFT-D2 or DFT-D3 functionals within that framework. To evaluate the influence of dispersion interactions, additional calculations were carried out using the DFT-D2 method in Quantum ESPRESSO. For K_2_O, the total energy changed from –267.74936184 Ry to -267.77910749 Ry, with a corresponding bandgap (E_g_) shift from 1.161 eV to 1.163 eV. Based on this negligible impact, vdW corrections were not included in the primary WIEN2k results. Nevertheless, future work should consider using more advanced vdW-corrected approaches for improved quantitative accuracy.

Table S1. represents the orbital contribution of the 1T-KXO compound.

| **Compound** | **Valence Band (VB) Major Orbital** | **Conduction Band (CB) Major Orbitals** |
| --- | --- | --- |
| 1T-K_2_O | O-2p | K-4s, 3d |
| 1T-KNaO | O-2p | K-4s > Na-3s, 3p |
| 1T-KRbO | O-2p | K-4s > Rb-5s, 4p |

To provide a more comprehensive discussion, the reduced mass ($m_{\mu}$), and exciton binding energy ($E_{ex}$) have been calculated using the formula,

$$\frac{1}{m_{\mu}}=\left( \frac{1}{m_{e}^{*}}+ \frac{1}{m_{h}^{*}} \right) (1)$$

$$E_{ex}= \frac{4m_{\mu}R_{\infty}}{m_{0}\varepsilon_{1}^{2}(0)} (2)$$

The calculated values of $m_{\mu}$, and $E_{ex}$ are tabulated in the Table S2. $\varepsilon_{1}(0)$ represents the static dielectric function, $R_{\infty}$ is the Rydberg constant, $m_{e}^{*}$ is the electron’s effective mass and $m_{h}^{*}$ is the hole’s effective mass.

Table S2. Represents the reduced mass and exciton binding energy of 1T-KXO monolayers.

| Compound | $m_{\mu}$ | $E_{ex} (meV)$ |
| --- | --- | --- |
| 1T-K_2_O | 0.24 $m_{e}$ | 4609 |
| 1T-KNaO | 0.24 $m_{e}$ | 4298 |
| 1T-KRbO | 0.25 $m_{e}$ | 4377 |

Table S3. Represents the values of $v_{t}$, $v_{l}$, $\theta_{a}$, $\gamma$, and $A$ of the 1T-KXO compound.

| **Compound** | $\boldsymbol{v}_{\boldsymbol{t}}$ | $\boldsymbol{v}_{\boldsymbol{l}}$ | $\boldsymbol{\theta}_{\boldsymbol{a}}$ | $\boldsymbol{\gamma}$ | $\boldsymbol{A}\boldsymbol{\times}\boldsymbol{10}^{\boldsymbol{-8}}$ |
| --- | --- | --- | --- | --- | --- |
| 1T-K_2_O | 2356.40 | 5391.95 | 184.17 | 2.43 | 3.29 |
| 1T-KNaO | 2646.04 | 5017.52 | 214.90 | 1.82 | 3.85 |
| 1T-KRbO | 2208.42 | 2210.40 | 149.92 | 0.5 | 2.15 |

Table S4. represents the data of predicted ZT using RF, LR and DFT values of 1T-KXO compounds.

| **Temperature (K)** | **1T-K_2_O** | | | **1T-KNaO** | | | **1T-KRbO** | | |
| --- | --- | --- | --- | --- | --- | --- | --- | --- | --- |
|  | **RF** | **LR** | **DFT** | **RF** | **LR** | **DFT** | **RF** | **LR** | **DFT** |
| 50 | 0.45 | 0.54 | 0.46 | 0.51 | 0.53 | 0.55 | 0.60 | 0.57 | 0.65 |
| 100 | 0.51 | 0.57 | 0.50 | 0.56 | 0.55 | 0.56 | 0.75 | 0.65 | 0.69 |
| 150 | 0.54 | 0.61 | 0.54 | 0.54 | 0.55 | 0.55 | 0.73 | 0.68 | 0.68 |
| 200 | 0.57 | 0.63 | 0.56 | 0.57 | 0.56 | 0.61 | 0.72 | 0.72 | 0.69 |
| 250 | 0.58 | 0.65 | 0.57 | 0.65 | 0.58 | 0.73 | 0.71 | 0.75 | 0.69 |
| 300 | 0.58 | 0.65 | 0.58 | 0.78 | 0.61 | 0.86 | 0.72 | 0.77 | 0.69 |
| 350 | 0.58 | 0.65 | 0.58 | 0.89 | 0.63 | 0.92 | 0.71 | 0.78 | 0.68 |
| 400 | 0.58 | 0.64 | 0.58 | 0.92 | 0.64 | 0.93 | 0.69 | 0.79 | 0.68 |
| 450 | 0.58 | 0.64 | 0.58 | 0.88 | 0.64 | 0.90 | 0.68 | 0.78 | 0.68 |
| 500 | 0.59 | 0.63 | 0.58 | 0.86 | 0.64 | 0.87 | 0.68 | 0.77 | 0.68 |
| 550 | 0.59 | 0.61 | 0.59 | 0.83 | 0.64 | 0.83 | 0.68 | 0.76 | 0.68 |
| 600 | 0.59 | 0.60 | 0.59 | 0.79 | 0.64 | 0.80 | 0.68 | 0.75 | 0.68 |
| 650 | 0.60 | 0.59 | 0.60 | 0.78 | 0.64 | 0.78 | 0.68 | 0.74 | 0.68 |
| 700 | 0.60 | 0.58 | 0.60 | 0.76 | 0.63 | 0.76 | 0.68 | 0.73 | 0.68 |
| 750 | 0.61 | 0.57 | 0.61 | 0.75 | 0.63 | 0.75 | 0.69 | 0.71 | 0.69 |
| 800 | 0.62 | 0.56 | 0.61 | 0.73 | 0.62 | 0.74 | 0.69 | 0.70 | 0.69 |
| 850 | 0.62 | 0.54 | 0.62 | 0.72 | 0.62 | 0.74 | 0.70 | 0.68 | 0.70 |
| 900 | 0.62 | 0.53 | 0.63 | 0.72 | 0.61 | 0.73 | 0.70 | 0.66 | 0.70 |
| 950 | 0.63 | 0.52 | 0.63 | 0.72 | 0.60 | 0.73 | 0.70 | 0.65 | 0.71 |
| 1000 | 0.64 | 0.50 | 0.64 | 0.71 | 0.60 | 0.72 | 0.69 | 0.63 | 0.71 |
